# Factors associated with patients’ and GPs’ assessment of the burden of treatment in multimorbid patients: a cross-sectional study in primary care

**DOI:** 10.1186/s12875-019-0974-z

**Published:** 2019-06-28

**Authors:** Lilli Herzig, Andreas Zeller, Jérôme Pasquier, Sven Streit, Stefan Neuner-Jehle, Sophie Excoffier, Dagmar M Haller

**Affiliations:** 1 Department of Family Medicine, General Medicine and Public Health Centre, University of Lausanne, Bugnon 44, 1011 Lausanne, Switzerland; 2 Centre for Primary Health Care, University of Basel, Basel, Switzerland; 3 Institute of Social and Preventive Medicine, Lausanne University Hospital, Lausanne, Switzerland; 4 Institute of Primary Health Care (BIHAM), University of Bern, Bern, Switzerland; 5 Institute of Primary Care, University and University Hospital of Zurich, Zurich, Switzerland; 6 Primary Care Unit, Faculty of Medicine, University of Geneva, Geneva, Switzerland; 7 Croisettes 14, 1066, Epalinges, Switzerland

**Keywords:** Chronic diseases, Multimorbidity, Primary care, Burden of treatment

## Abstract

**Background:**

Multimorbid patients may experience a high burden of treatment. This has a negative impact on treatment adherence, health outcomes and health care costs. The objective of our study was to identify factors associated with the self-perceived burden of treatment of multimorbid patients in primary care and to compare them with factors associated with GPs assessment of this burden.

**Method:**

A cross sectional study in general practices, 100 GPs in Switzerland and up to 10 multimorbid patients per GP. Patients reported their self-perceived burden of treatment using the Treatment Burden Questionnaire (TBQ, possible score 0–150), whereas GPs evaluated the burden of treatment on a Visual Analog Scale (VAS) from 1 to 9. The study explored medical, social and psychological factors associated with burden of treatment, such as number and type of chronic conditions and drugs, severity of chronic conditions (CIRS score), age, quality of life, deprivation, health literacy.

**Results:**

The GPs included 888 multimorbid patients. The overall median TBQ was 20 and the median VAS was 4. Both patients’ and GPs’ assessment of the burden of treatment were inversely associated with patients’ age and quality of life. In addition, patients’ assessment of their burden of treatment was associated with a higher deprivation score and lower health literacy, and with having diabetes or atrial fibrillation, whereas GPs’ assessment of this burden was associated with the patient having a greater number of chronic conditions and drugs, and a higher CIRS score.

**Conclusion:**

Both from patients’ and GPs’ perspectives TB appears to be higher in younger patients. Whereas for patients the burden of treatment is associated with socio-economic and psychological factors, GPs’ assessments of this burden are associated with medical factors. Including socio-economic and psychological factors on patients’ self-perception is likely to improve GPs’ assessments of their patients’ burden of treatment thus favoring patient-centered care.

**Electronic supplementary material:**

The online version of this article (10.1186/s12875-019-0974-z) contains supplementary material, which is available to authorized users.

## Background

Multimorbidity (commonly defined as having two or three chronic conditions) is increasing in prevalence and becoming a major health concern worldwide. [1–3] It is associated with a higher burden of disease, poorer health outcomes and reduced quality of life, more frequent hospital admissions, a higher number of provider visits, higher mortality and increasing healthcare costs. [4–8] Multimorbity is also associated with polypharmacy and its potentially negative consequences on safety of care (e.g. drug interactions). [9, 10] The prevalence of multimorbidity is high in primary care (PC) and these patients require long-term care. [11]

Physicians increasingly encourage multimorbid patients to develop self-management skills. For patients this involves finding time for health education, lifestyle changes, self-assessment and monitoring. Self-management in the context of multimorbidity has important implications for the organization of patients’ daily lives. [12, 13] In other words, multiple chronic conditions are associated with the “work to be patient”, also called burden of treatment (which is not the same as the burden of diseases). [14, 15] Burden of treatment has an impact on adherence to treatment. The higher the burden of treatment, the higher the need for patients to invest time in acquiring knowledge about their diseases and their relevant treatment options. [12, 16, 17]

Burden of treatment is a recent concept, initially introduced for single chronic conditions, and then for single chronic conditions in combination with co-morbidities. [18] It is not defined very clearly, and there are considerable variations between authors, studied populations and countries. [19, 20] To date, most studies on burden of treatment are qualitative in nature, and include the study of different aspects of daily life such as financial burden, lack of knowledge, time spent for diet and exercises, medication burden and frequent healthcare appointments. [19, 21] In 2012, based on these qualitative studies, Tran et al. proposed a quantitative measure to assess patients’ self-perceived burden of treatment: the Treatment Burden Questionnaire (TBQ). [14]

Little evidence is available in relation to the factors associated with the burden of treatment for multimorbid patients in primary care. Some studies have identified physical, financial, time and psychosocial factors [19, 22, 23]. Different studies also suggest an association with socio-economic determinants and social deprivation. [16] The association between patient characteristics and burden of treatment has not been explored before. Knowledge of these factors could guide GPs in identifying patients exposed to a higher burden of treatment.

Therefore, the aim of our study was to describe the medical, psychological and socio-economic factors associated with patients’ self-perceived burden of treatment. A secondary aim was to compare these factors with those associated with GPs’ assessment of their patients’ burden of treatment, in order to formulate hypotheses about the extent to which GPs’ assessment of this burden might favor patient-centered care.

## Method

Our analyses are based on the cross-sectional study “Multi-Morbidity in Family Medicine” (MMFM). The detailed study protocol and the first results have been published elsewhere. [24, 25] Briefly, MMFM included 888 multimorbid patients in primary care and involved a convenience sample of 100 GPs across five large regions in Switzerland. Eligible participants were multimorbid patients aged over 18 years old, suffering from at least three chronic conditions identified in a predefined list of 75 items based on the International Classification of Primary Care 2, (ICPC-2). [26] Each included patient gave his or her written informed consent. GPs completed a written form, whereas patients answered a standardized telephone interview conducted by trained research collaborators. Interviews were conducted in French or German depending on the region of Switzerland in which the patient lived. [24, 25]

### Ethical approval

The Human Research Ethics Committee of the Canton of Vaud, acting as the lead ethics committee for Switzerland (Protocol 315/14), approved the protocol.

We evaluated the burden of treatment from two perspectives:

### From the patient’s perspective

We used the above-mentioned validated questionnaire, the TBQ. [14] The TBQ was first published and validated in French. We followed standard steps to create a German version: parallel translation by two professional translators, consolidation and back translation. [27] We chose the TBQ in 2013, when the protocol was established, because it was then the only existing score. We used 15 items-version of the French validation. (Additional file 1). The TBQ-score is computed by simply adding patients’ answers for each item on a 10-point Likert scale. Consequently, the TBQ score ranges between 0 (no burden) and 150 (highest burden).

### From the GP’s perspective

GPs estimated the burden of treatment for each patient they had included on a VAS scale from 1 to 9 where 1 is the lowest and 9 the highest burden.

#### In addition, the following variables were analyzed

Patient’s age and gender, the number of chronic conditions and drugs (as reported by GPs), the severity of chronic conditions as assessed by GPs on the Cumulative Illness Rating Scale (CIRS) [28], patients’ quality of life (EQ. 5D 3 L) [29], their health literacy (HL score) [30], level of deprivation (DipCare score) [31]. We also examined the association between the burden of treatment and the presence of specific chronic conditions, choosing conditions that had a prevalence > 20% in our sample: hypertension (ICPC2 codes K85,86), cardiovascular risk factors (K22), diabetes (T89, 90), obesity (T82) ischemic heart diseases (K 74, 76), depression (P76) osteoarthritis of the knee (L90), general pain (A01) and atrial fibrillation (K78). (Also see results Table 1). We choose a cut-off of 20% to include the chronic conditions in the regression model in order to limit the number of variables to include with the aim to avoid overfitting.

**Table 1:** Characteristics of the 888 included patients and main results regarding associated factors

Description	Type	N missing values	Value	Frequency*	Percentage*
TBQ score (patient perspective)	Numeric	0	Mean/SD	26.8	18.6
			Median/IQR	20.0	[15.0; 33.0]
log(TBQ score + 1) (patient perspective)	Numeric	0	Mean/SD	3.2	0.6
			Median/IQR	3.0	[2.8; 3.5]
VAS rating (GP perspective)	Numeric	1	Mean/SD	4.5	1.7
			Median/IQR	4.0	[3.0;6.0]
Patient’s age	Numeric	0	Mean/SD	72.9	12.0
			Median/IQR	74.2	[65.9; 81.8]
Patient’s gender is male	Logical	0	TRUE	428	48.2
Location of practice	Category	0	Urban	383	43.1
			Semi-urban	361	40.7
			Rural	144	16.2
Marital status	Category	0	Single	85	9.6
			Married	437	49.2
			Separated/ divorced	150	16.9
			Widow	216	24.3
Highest level of schooling achieved	Category	1	Compulsory education	195	22.0
			Upper secondary level	337	38.0
			Tertiary level	355	40.0
Number of CC (GP’s assessment)	Numeric	4	Mean/SD	7.2	2.9
			Median/IQR	7.0	[5.0;8.2]
CIRS	Numeric	1	Mean/SD	1.7	0.4
			Median/IQR	1.7	[1.5–2.0]
Total number of different pharmacological treatment the patient is currently taking	Numeric	0	Mean/SD	7.7	3.5
			Median/IQR	7.0	[5.0;10.0]
Use of a pillbox (dispenser)	Logical	0	TRUE	405	45.6
Home-based care (patient report)	Logical	10	TRUE	94	10.6
Total EQ-5D-3 L score	Numeric	0	Mean/SD	69.9	17.6
			Median/IQR	70.5	[62.4;78.3]
EQ-5D-3 L health scale (VAS, 0–100)	Numeric	0	Mean/SD	63.2	19.3
			Median/IQR	65.0	[50.0;80.0]
Deprivation score (DipCare)	Numeric	0	Mean/SD	1.2	0.9
			Median/IQR	0.9	[0.5;1.8]
Health literacy score (HLS EU 6)	Numeric	577	Mean/SD	2.9	0.5
			Median/IQR	2.8	[2.7;3.2]
Hypertension (ICPC:K85,86)	Logical	0	TRUE	657	74.0
Cardiovascular risk factors (ICPC:K22)	Logical	0	TRUE	391	44.0
Diabetes (ICPC: T89,90)	Logical	0	TRUE	277	31.2
Obesity (ICPC: T82)	Logical	0	TRUE	274	30.9
Ischemic heart disease (ICPC: K74,76)	Logical	0	TRUE	258	29.1
Depression (ICPC: P76)	Logical	0	TRUE	228	25.7
Knee osteoarthritis (ICPC:L90)	Logical	0	TRUE	223	25.1
Pain general (ICPC:A01)	Logical	0	TRUE	198	22.3
Atrial Fibrillation (ICPC:K78)	Logical	0	TRUE	195	22.0

### Statistical analyses

We conducted descriptive analyses, presented as mean, standard deviation, median and interquartile range for quantitative variables and as frequencies and proportions for categorical variables.

Univariate and multivariate linear regressions were conducted to determine which factors were associated with our two outcomes, i.e. TBQ scores for the patient and VAS scores for GPs. As the TBQ was asymmetrically distributed in our sample, a logarithmic transformation of the latter was considered as the dependent variable in the regression. The GP-cluster effect was introduced into the model as a random intercept. Multiple imputations by fully conditional specification were used to handle missing values. Each missing value was imputed 15 times. To avoid overfitting, we proceeded to a forward selection of the independent variables in both multivariable regressions (TBQ and VAS). At each step of the selection, for each new variable to be included, the null hypothesis was tested that the extra parameter was zero. The variable corresponding to the smallest *p*-value was included in the model. The selection procedure was interrupted when no p-value was lesser or equal to 0.1. As the research team suspected an association between the number of chronic conditions, the number of drugs and the CIRS (calculated as the ratio of total score/number of endorsed categories) and the burden of treatment, these variables were included in the model before starting the forward selection procedure. Finally, we computed the variance inflation factor (VIF) of the selected variables to ensure their non-collinearity.

#### Dependent variables

Log (TBQ score + 1), VAS scale.

#### Independent variables

All variables described in Table 1, except for the two dependent variables.

All analyses were conducted using R version 3.4.4 and the package mice version 2.46 for the imputation of missing values. [32, 33]

## Results

We included 888 patients (mean age of 72.9 years, 48% were men). They had a mean of 7.2 chronic conditions (SD 2.9) and were taking a mean of 7.5 pills a day (SD 3.5). Patients’ characteristics and factors associated with the TBQ (Mean (SD), Median (IQR), Frequency and Percentage) are listed in Table 1. We created a correlation matrix, which can be found in the additional file (Additional file 2).

### Patients’ perspective

**Fig. 1:**
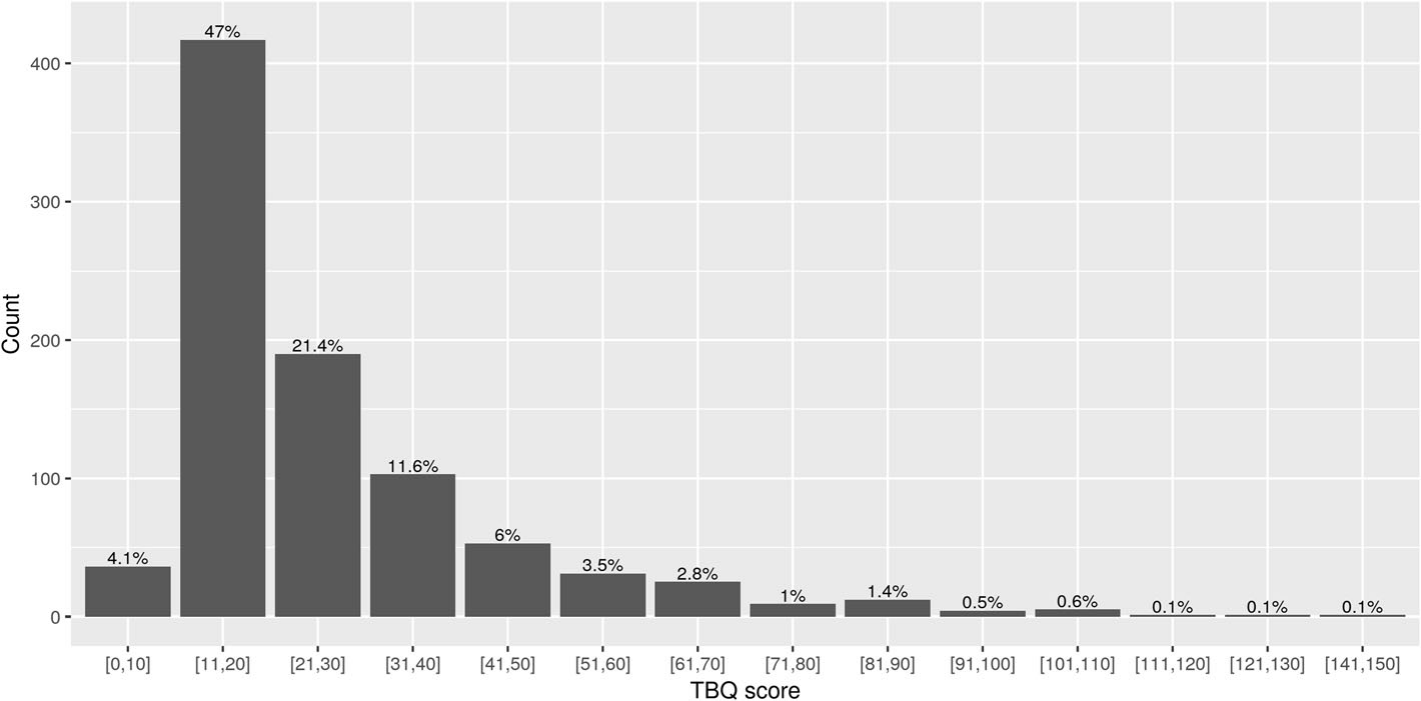
Overall TBQ score

**Fig. 2:**
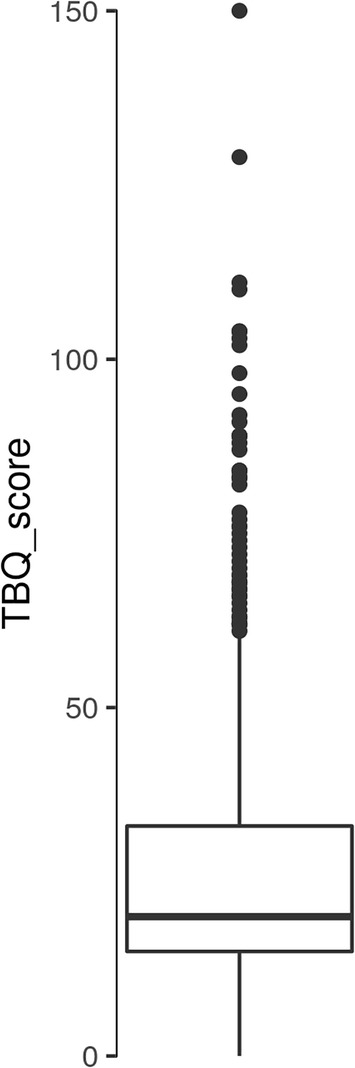
TBQ score Barblot

The overall median TBQ score was 20 (Q25% = 15, Q75% = 33). The distribution is shown in Figs. 1 and 2.

The results of the regressions are shown in Table 2. The effect size is given on the logarithmic scale. The forward selection process led to the inclusion of age, education, use of a pillbox, quality of life (EQ5D3L_score and EQ5D3L VAS), DipCare index, health literacy, diabetes and atrial fibrillation in the multivariable model (as previously stated, number of chronic conditions, CIRCS (ratio total score/number of endorsed categories) and number of drugs were included in the model before the selection). Variance inflation factors were relatively low. There is thus no major collinearity between the variables introduced in the model. Younger age, higher education, higher deprivation score, lower quality of life and health literacy scores were significantly associated with higher TBQ scores. Furthermore, patients with diabetes or atrial fibrillation had higher TBQ scores. We found no association between depression (P76), general pain (A01) or the CIRCS, and TBQ scores.

**Table 2:** Univariate and multivariate regression of log(TBQ + 1) with medical, social and psychological factors (patients’ perspective)

Description		Unvariate regressions		Multivariate regression		
		Beta coefficient (95% CI)	p-value	Beta coefficient (95% CI)	p-value	VIF*
Patient’s age in years		−0.019 (− 0.022;-0.016)	< 0.001	−0.017 (− 0.020;-0.014)	< 0.001	1.174
Patient’s gender is male		0.011 (− 0.064;0.085)	0.777			
Location of practice (ref: Urban)	**Semi-urban/suburban**	−0.058 (− 0.154;0.038)	0.236			
	**Rural**	−0.020 (− 0.148;0.107)	0.758			
Marital status (ref: Single)	**Married**	− 0.192 (− 0.319;-0.065)	0.003			
	**Separated/divorced**	−0.009 (− 0.155;0.136)	0.902			
	**Widow**	−0.362 (− 0.499;-0.225)	< 0.001			
Highest level of schooling achieved (ref: Compulsory education)	**Upper secondary level**	−0.036 (− 0.134;0.063)	0.480	− 0.023 (− 0.103;0.058)	0.584	1.056
	**Tertiary level**	0.093 (− 0.005;0.191)	0.062	0.115 (0.034;0.196)	0.006	1.056
GP assessment of number of chronic conditions		0.019 (0.005;0.033)	0.006	0.004 (−0.008;0.016)	0.551	1.265
CIRCS		0.200 (0.101;0.299)	< 0.001	0.033 (−0.049;0.115)	0.425	1.076
Total number of different pharmacological treatment the patient is currently taking		0.030 (0.019;0.041)	< 0.001	0.008 (−0.003;0.018)	0.160	1.471
Use of a pillbox (dispenser)		0.132 (0.058;0.206)	< 0.001	0.076 (0.012;0.139)	0.020	1.122
Home-based care (patient report)		0.015 (−0.105;0.135)	0.807			
Total EQ-5D-3 L score		−0.012 (− 0.014;-0.010)	< 0.001	− 0.005 (− 0.007;-0.003)	< 0.001	1.717
EQ-5D-3 L health scale (VAS, 0–100)		− 0.010 (− 0.012;-0.009)	< 0.001	−0.004 (− 0.006;-0.003)	< 0.001	1.457
Deprivation score (DipCare)		0.196 (0.159;0.234)	< 0.001	0.060 (0.022;0.097)	0.002	1.399
Health literacy score (HLS EU 6)		−0.329 (− 0.412;-0.245)	< 0.001	− 0.162 (− 0.237;-0.087)	< 0.001	1.167
Hypertension		− 0.118 (− 0.203;-0.034)	0.006			
Cardiovascular risk		−0.022 (− 0.098;0.054)	0.570			
Diabetes		0.099 (0.019;0.178)	0.016	0.107 (0.039;0.174)	0.002	1.098
Obesity		0.067 (−0.014;0.147)	0.105			
Ischemic heart disease		−0.077 (− 0.159;0.004)	0.063			
Depression		0.210 (0.126;0.294)	< 0.001			
Knee osteoarthritis		0.011 (−0.075;0.097)	0.802			
General pain		0.177 (0.088;0.266)	< 0.001			
Atrial fibrillation		−0.058 (−0.147;0.032)	0.209	0.105 (0.028;0.183)	0.008	1.151

* variance inflation factor to exclude co-linearity

### GPs’ perspective

**Fig. 3:**
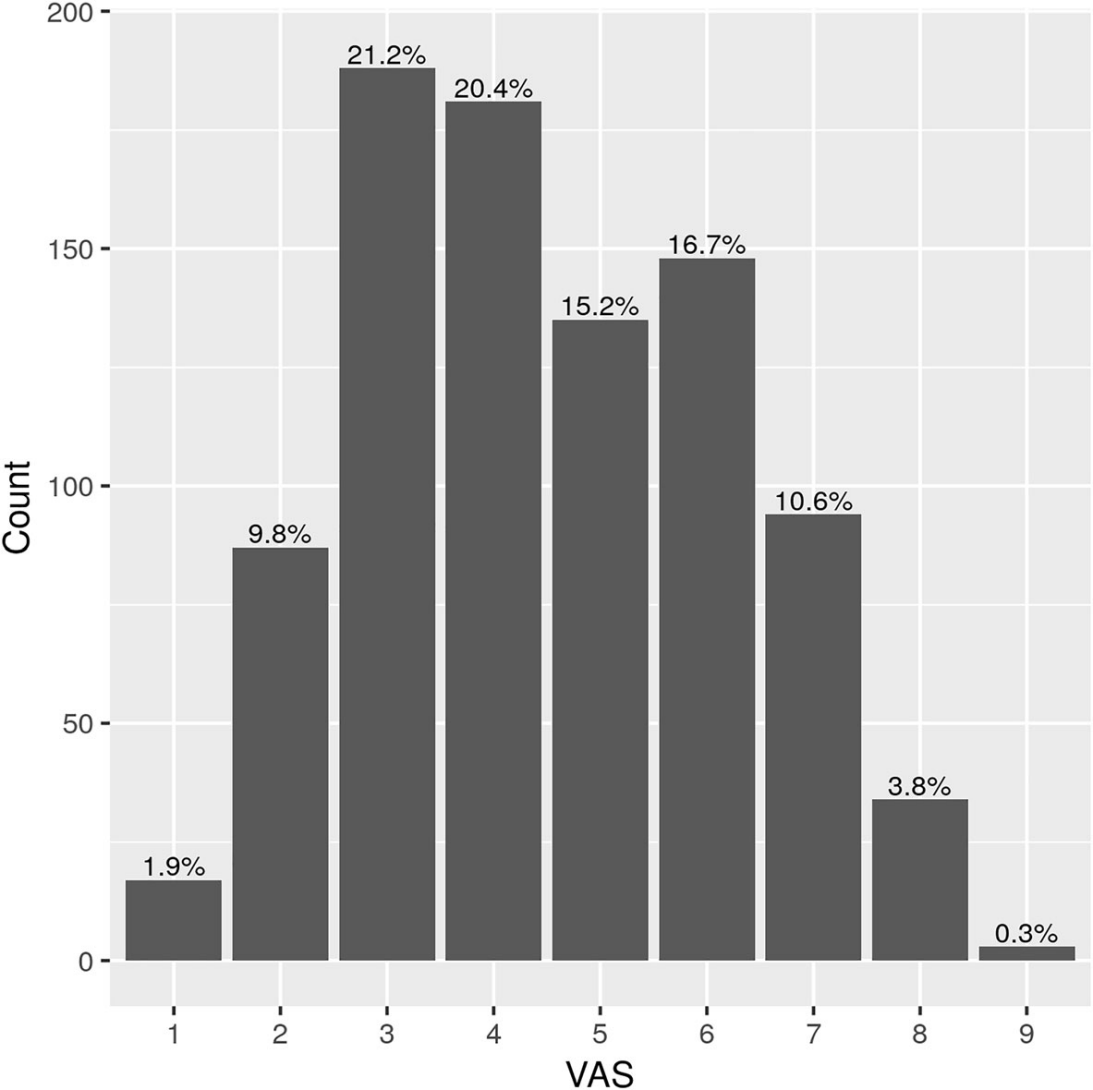
VAS (GPs perspective) distribution

The median VAS rating was 4 (Q25% = 3, Q75% = 6). The results of the regressions are shown in Table 3 and the distribution is shown in Fig. 3. Forward selection resulted in the inclusion of age, use of a pillbox, home-based care, quality of life (eq5d3l score and eq5d3l VAS), health literacy, general pain and atrial fibrillation in the multivariable model (again, number of chronic conditions, CIRS and number of drugs were included in the model before the selection). We found an association between a higher VAS rating and lower age, higher number of chronic conditions and drugs, higher CIRCS and a lower quality of life score.

**Table 3:** Univariate and multivariate regression of VAS with medical, social and psychological factors (GPs’ perspective)

Description	Value	Unvariable regressions		Multivariable regression		
		Beta coefficient (95% CI)	p-value	Beta coefficient (95% CI)	p-value	VIF*
Patient’s age in years		−0.014 (− 0.023;-0.005)	0.002	−0.015 (− 0.023;-0.007)	< 0.001	1.185
Patient’s gender		−0.213 (− 0.428;0.002)	0.052			
Location of practice (ref: Urban)	**Semi-urban/suburban**	0.000 (−0.417;0.417)	1.000			
	**Rural**	0.065 (−0.491;0.620)	0.820			
Marital status (ref: Single)	**Married**	−0.226 (− 0.602;0.149)	0.237			
	**Separated/divorced**	0.110 (−0.318;0.538)	0.614			
	**Widow**	−0.211 (− 0.614;0.193)	0.306			
Highest level of schooling achieved (ref: Compulsory education)	**Upper secondary level**	−0.116 (− 0.402;0.171)	0.428			
	**Tertiary level**	−0.123 (− 0.408;0.162)	0.398			
GP assessment of number of chronic conditions		0.165 (0.124;0.206)	< 0.001	0.069 (0.031;0.107)	< 0.001	1.239
CIRCS		1.765 (1.488;2.042)	< 0.001	1.267 (1.015;1.520)	< 0.001	1.088
Total number of different pharmacological treatment the patient is currently taking		0.196 (0.165;0.227)	< 0.001	0.102 (0.070;0.134)	< 0.001	1.380
Use of a pillbox (dispenser)		0.595 (0.383;0.807)	< 0.001	0.136 (−0.045;0.317)	0.142	1.106
Home-based care (patient report)		0.964 (0.623;1.305)	< 0.001	0.336 (0.041;0.631)	0.026	1.133
Total EQ-5D-3 L score		−0.036 (−0.042;-0.031)	< 0.001	−0.016 (− 0.022;-0.010)	< 0.001	1.681
EQ-5D-3 L health scale (VAS, 0–100)		−0.026 (− 0.031;-0.020)	< 0.001	−0.005 (− 0.010;0.000)	0.056	1.393
Deprivation score (DipCare)		0.496 (0.385;0.607)	< 0.001			
Health literacy score (HLS EU 6)		−0.612 (−0.855;-0.369)	< 0.001	− 0.176 (− 0.382;0.030)	0.093	1.133
Hypertension		−0.150 (− 0.396;0.096)	0.231			
Cardiovascular risk		−0.225 (− 0.453;0.002)	0.052			
Diabetes		−0.047 (− 0.279;0.185)	0.691			
Obesity		−0.027 (− 0.261;0.207)	0.823			
Ischemic heart disease		0.156 (−0.079;0.392)	0.193			
Depression		0.673 (0.429;0.916)	< 0.001			
Knee osteoarthritis		0.192 (−0.060;0.445)	0.136			
General pain		0.794 (0.534;1.055)	< 0.001	0.198 (−0.029;0.425)	0.087	1.135
Atrial fibrillation		0.287 (0.024;0.549)	0.032	0.233 (0.009;0.457)	0.042	1.135

* variance inflation factor to exclude co-linearity

## Discussion

Our study analyzed factors associated with multimorbid patients’ self-perceived burden of treatment as well as factors associated with GPs’ assessment of this burden. Younger, educated patients, those with a lower quality of life, a higher deprivation score and lower health literacy reported higher self-perceived burden of treatment. Suffering from diabetes or atrial fibrillation was also independently associated with higher patients’ self-reported burden of treatment. From the GPs’ perspective, burden of treatment was also perceived to be higher in younger patients and in those suffering from atrial fibrillation. Medical factors (number of chronic conditions, number of drugs…) rather than social factors (deprivation, literacy…) appeared to be more strongly associated with GPs’ perceptions of their patients’ burden of treatment. GPs seemed to integrate the notion of deprivation or lower health literacy in their estimation of their patients’ burden of treatment only to a minor degree.

The most important finding in our study is the association between higher burden of treatment and younger age. We hypothesize that this may be due to a higher impact of multimorbidity on an active professional life: younger patients may have more difficulties integrating the workload of treatment for several chronic conditions into an active professional life. Alternatively, older patients’ self-perceived burden of treatment may be lower due to a higher acceptance of their chronic conditions with time, or as a consequence of their social education (readiness to accept a negative condition). Most previous studies on multimorbidity were conducted in older populations only and this may be the reason why our finding is new. [1, 34, 35].

A second important finding is the difference in factors associated with burden of treatment between patients and GPs. While patients’ estimates of their burden of treatment were predominantly associated with psychological and socioeconomic factors (deprivation, health literacy and lower quality of life), GPs’ perspectives were more strongly associated with medical factors such as the number of chronic conditions and drugs. In order to achieve realistic goals, practice patient-centered care and apply shared decision-making care models - as proposed by Muth et al. - GPs should probably explore their patients’ self-perceived burden of treatment, rather than rely only on their own assessment. [36, 37] Our results are in line with other publications showing that patient-provider concordance needs to be improved. [38, 39]

Caring for multimorbid patients is a complex task which needs an understanding beyond the simple compilation of chronic conditions or bio-medical concepts. [40] However, our study shows that GPs estimates of the burden of treatment most strongly relate to such factors. As multimorbidity is a long-term challenge and needs a paradigm change “from cure to care” or “from guidelines to mindlines” the integration and correct estimation of patients’ burden of treatment is important. [11, 41] This includes patient’s ability for self-management and an understanding of factors that may limit this ability, such as low health literacy. [42, 43] Our study shows the importance of patient-centered care: the role of GPs is to explore patients’ burden and limits, including socio-economic and psychological factors.

An important challenge is to improve treatment adherence in multimorbid patients. This has an impact on long-term outcomes and health care costs. Indeed, higher burden of treatment is associated with poorer treatment adherence. [18, 44] Therefore, a better understanding of factors associated with patient’s ability and workload is likely to help improve treatment adherence. [40] We need to “start treatment for patients not for diseases” as proposed by May. [45] When starting new treatments, we also need to weigh the added burden of treatment against the (sometimes-small) effects of these new treatments in multimorbid patients. Therefore, for every potential treatment (e.g. recommended by a guideline), GPs should consider the potential added burden of treatment and discuss and weigh this with their patients. Evidence from the literature confirms that better knowledge of patients’ needs and goals, improved relationship, patient-centered care and shared decisions between GPs and patients improve treatment adherence and patients’ satisfaction as well as outcomes. [17, 46–48] Therefore, it seems essential to integrate individual patients’ perceived burden of treatment into every decision about the long-term management in the context of multimorbidity.

Another finding of our study suggests that burden of treatment is particularly high for diabetic patients. Yet the literature on diabetes rarely integrates burden of treatment factors such as emotional elements, diet or food constraints, which are highly important for patients with diabetes. [17, 49] We hypothesize that the burden of treatment is particularly high for patients with diabetes because the treatment requires activities in multiple domains, e.g. daily serum glucose controls, adaptation of diet, physical exercise. This has an important impact on social life and attitudes.

Atrial fibrillation was also associated with a higher burden of treatment. This might be explained by the need for anticoagulation, which has an impact on drug and food interactions or on the number of GP visits required.

### Strengths

Our study is one of the first quantitative studies to use Tran’s TBQ score in a large population of multimorbid patients in primary care. Furthermore, to the best of our knowledge it is the first study to integrate factors associated with burden of treatment both from GPs’ and from patients’ perspectives.

Another strength is the inclusion of younger patients in our sample. Most studies on multimorbidity usually only select older patients. And our study covers a diverse sample from French and German-speaking regions, recruited over most of an entire country, thus better reflecting usual care in multimorbid patients in primary care.

Finally, our study assessed an important range of social as well as medical variables potentially associated with burden of treatment in multimorbid patients in PC, thus covering a wide range of dimensions of the burden of treatment.

### Limitations

Our study has some limitations. First the French-version of the TBQ, developed in 2012, has not been used elsewhere. We created a German version of the TBQ using careful translation-back-translation of the validated French version, but did not validate this version per-se. Furthermore, the original TBQ was developed and validated for face to face interviews and we cannot exclude some differences when using phone interviews. Because the TBQ was not developed for this purpose, and also to limit the study burden for GPs, we used a VAS to assess burden of treatment from the GPs perspective and not the TBQ. This may have had an influence on the comparison between the two perspectives. In addition, this method precluded any direct comparison between GPs’ and patients’ TBQ scores. Finally, there were many missing values on the health literacy score. We used multiple imputations to adjust for this, which may have influenced our findings.

## Conclusions

Both from patients’ and GPs’ perspectives burden of treatment appears to be higher in younger patients. Whereas for patients burden of treatment is associated with socio-economic and psychological factors, GPs assessment appears to be associated with medical factors such as number or severity of chronic conditions and number of drugs. These findings offer new guidance to improve patient-centered care. Indeed, including socio-economic and psychological factors or relying on patients’ self-perception is likely to improve GPs’ assessments of their multimorbid patients’ burden of treatment. A more adequate estimate of this burden may help GPs weigh the benefits of any added treatment against the risk of adding to this burden thus threatening adherence to care. Further research should identify pragmatic ways of integrating the assessment of patients’ burden of treatment in the routine care of multimorbid patients seen in primary care.

## Additional files

Additional file 1: TBQ French and German version. (DOCX 20 kb)

Additional file 2: Correlation matrix. (CSV 9 kb)

## Data Availability

Data are available at the Family Medicine Institute of Lausanne.

## Abbreviations

CIRCS: Cumulative illness rating scale
GP: General practitioner
PC: Primary care
TBQ: Treatment burden questionnaire
VAS: Visual analog scale
