# Supplementary material for: Factors associated with patients’ and GPs’ assessment of the burden of treatment in multimorbid patients: a cross-sectional study in primary care
Source: BMC Fam Pract. 2019 Jun 28;20:88. doi: 10.1186/s12875-019-0974-z (PMC6598361; doi:10.1186/s12875-019-0974-z)
Supplement: Supplementary file 1 — TBQ French and German version. (DOCX 20 kb) [file 12875_2019_974_MOESM1_ESM.docx]

Additional file 1

**The 15 items of the TBQ (French and German Version)**

**Questions:**

How do you evaluate:

**I A.** The taste, shape or size of your tablets and/or the inconvenience caused by your injections (for example, pain, bleeding, scars)

**1B.** The number of times you have to take your medication every day

**1C.** The things you do to remind yourself to take your daily medication and/or to manage your treatment when you are not at home

**1D.** The specific conditions when taking your medication (for example, taking it at a specific time of the day or meal, not being able to do certain things after taking them like driving or lying down)

**IE.*** The specific storing conditions of your medication (for example temperature)

**2A.** Lab tests and other exams (frequency, time spent and inconvenience of these exams)

**2B.** Self-monitoring (for example, taking your blood pressure or measuring your blood sugar yourself: frequency, time spent and inconvenience of this surveillance)

**2C.** Doctors visits (frequency and time spent for the visits)

**2D.** Arrange appointments and schedule doctors visits and lab tests

**3 A.** How would you rate the burden associated with taking care of paperwork from health insurance agencies, welfare organizations, hospitals and/or social care?

**3 B.*** How would you rate the burden associated with financial problems (i.e payment of treatments that are not covered by the insurance)

**4.** How would you rate the constraints associated with your diet (for example, not being allowed to eat certain foods)?

**5.** How would you rate the burden associated with the recommendations from your doctors to practice regular physical exercises?

**6.** What is the impact of your healthcare on your social relationships (for example, need for assistance, being ashamed to take your medication in front of people)?

**7.** Frequent healthcare reminds me of my health problems

*: *question 1 E and 3 B do not exist in the English version but only in the original French version used for our study*

**French version TBQ :**

Concernant la prise de vos médicaments, comment évalueriez vous les contraintes liées :

**1.A** Au goût, à la forme, à la taille de vos comprimés et aux désagréments causés par vos injections (douleur, saignements, séquelles inesthétiques…)?

**1.B** Au nombre de fois ou vous devez prendre vos médicaments par jour

|  | **1 C** Aux efforts que vous devez faire pour ne pas oublier de prendre vos médicaments, vous organiser pendant vos voyages, utiliser un pilulier… ?  **1 D** Aux précautions d’emploi de vos médicaments (Devoir prendre les médicaments à un moment précis de la journée ou du repas ; ne pas pouvoir faire certaines choses après les avoir pris comme s’allonger ou conduire…) ?  **1 E** Aux précautions que vous devez prendre pour conserver vos médicaments (au froid dans votre réfrigérateur…) ?  **2 A** Aux examens complémentaires (Analyses de sang, examens de radiologie…) : au nombre, au temps consacré et à la pénibilité de ces examens ?  **2 B** A la surveillance que vous réalisez vous même (Mesurer votre tension à domicile, faire des glycémies capillaires, tenir un carnet de suivi…) : à la fréquence, au temps consacré et à la pénibilité de cette surveillance **?**  **2C** Aux visites chez vos médecins : au nombre et au temps consacré à ces consultations ?  **2 D** A la prise de rendez-vous médicaux (Visites chez vos médecins, analyses de sang, autres examens…) et l’organisation de votre emploi du temps à cause de ces rendez-vous ?  **3 A** Les contraintes administratives liées à votre maladie (Formalités liées aux hospitalisations, aux remboursements par l’Assurance Maladie, aux démarches sociales…) ?  **3 B** Les contraintes financières liées à la maladie (par exemple : Prises en charge ou traitements non remboursés…) ?  **4** Comment évalueriez-vous les contraintes liées à votre régime (éviter certains aliments…) ?  **5** Comment évalueriez-vous la contrainte que représentent les recommandations de vos médecins pour faire des activités physiques (marche, course, natation…) ?  **6** Quel impact votre traitement a-t-il sur vos relations avec les autres (Avoir besoin d’être aidé dans la vie de tous les jours, avoir honte de prendre vos médicaments…) ?  **7**Le fait de me soigner régulièrement me rappelle que je suis malade » |  |
| --- | --- | --- |
|  | | |

|  |
| --- |

**German Version TBQ :**

In Bezug auf die Einnahme Ihrer Medikamente, wie würden Sie folgende damit verbundene Unannehmlichkeiten einschätzen:

**1A** Geschmack, Form, Größe der Tabletten und Unannehmlichkeiten ausgelöst durch Injektionen (Schmerzen, Blutungen, unästhetische Folgeerscheinungen…)?

**1B** Häufigkeit der täglichen Einnahme Ihrer Medikamente?

**1 C** Aufwand, die Einnahme Ihrer Medikamente nicht zu vergessen, der Organisation während einer Reise, des Benutzens einer Tablettenbox…?

**1 D** Vorsichtsmassnahmen zur Medikamenteneinnahme (zu einem bestimmten Zeitpunkt des Tages oder während des Essens; Einschränkungen nach der Einnahme der Medikamente wie sich Hinlegen oder nicht Autofahren…)?

**1 E** Vorkehrungen zur korrekten Aufbewahrung der Medikamente (im Kühlschrank…)?

**2 A** Zusätzliche Untersuchungen (Blutanalysen, bildgebende Verfahren…): Häufigkeit, Zeitaufwand und Beschwerlichkeit dieser Untersuchungen?

**2 B** Persönliche Überwachung (Blutdruckmessung zu Hause, Blutzuckerbestimmung, Buchführung…): Häufigkeit, Zeitaufwand und Beschwerlichkeit der Maßnahmen?

**2 C** Arztbesuche: Häufigkeit und Zeitaufwand der Termine?

**2D** Festlegen der Termine (Arzttermine, Blutanalysen, weitere Untersuchungen…) und Organisation Ihres Tagesablaufs aufgrund dieser Termine?

**3 A** Administrativer Aufwand im Zusammenhang mit Ihrer Krankheit (Formalitäten in Bezug auf eine Krankenhausaufnahme, Rückerstattung durch die Krankenkasse, Anträge an soziale Träger…)?

**3 B** Finanzielle Aufwendungen im Zusammenhang mit Ihrer Krankheit (zum Beispiel: Nichterstattung von Pflege und Behandlungen…)

**4** Einschränkungen im Zusammenhang mit **Ihrer Ernährung** (Vermeiden bestimmter Nahrungsmittel…)?

**5** Unannehmlichkeiten durch ärztliche Empfehlungen hinsichtlich körperlicher Aktivitäten (Gehen, Laufen, Schwimmen…)?

**6** Welche Auswirkungen hat Ihre Behandlung auf Ihr Verhältnis zu anderen Menschen (Bedarf von Hilfe im Alltag, Scham aufgrund der Medikamenteneinnahme…)?

**7** «Die Tatsache der regelmäßigen Behandlung erinnert mich daran, dass ich krank bin»
